# Modeling of Polymer Friction on Boundaries of Solids and Inside Materials

**DOI:** 10.3390/ma14206187

**Published:** 2021-10-18

**Authors:** Alfred Zmitrowicz

**Affiliations:** Institute of Fluid-Flow Machinery, Polish Academy of Sciences, ul. J. Fiszera 14, 80-231 Gdańsk, Poland; azmit@imp.gda.pl; Tel.: +48-58-5225-134

**Keywords:** anisotropy, heterogeneity, dry friction, Langevin equation, viscous friction

## Abstract

Friction models are proposed for anisotropic and heterogeneous dry friction on boundaries of polymer solids. Unit vectors and oriented angles of sliding velocities, radii of curvature and unit normal vectors of sliding trajectories are taken as independent variables in constitutive equations of anisotropic and heterogeneous friction. Heterogeneous dry friction of a polymer pin in pin-on-disc tests is illustrated in the case of Archimedean spiral trajectory. Individual molecular chains composing polymer materials can move inside the material with a high degree of friction anisotropy. The resistance of macromolecule motion is considered with respect to micromechanical models of macromolecules, their kinematics, and friction laws. Two approaches are applied for modeling of anisotropic friction inside polymer materials: continuum-based models (anisotropic viscous friction) and micromechanical models (anisotropic dry friction). Examples of macromolecule dry friction are considered under conditions of spinning and sliding of a disc-like macromolecule and snake-like sliding of a long macromolecule.

## 1. Introduction

This study is an extension of the research presented at 14th World Congress on Computational Mechanics (WCCM) and European Community on Computational Methods in Applied Sciences (ECCOMAS) Congress 2020, virtual congress: 11–15 January 2021 [1].

Polymers and polymer composites are materials of great importance in friction assemblies of machines and devices operating in unlubricated contact conditions (or in starved lubrication conditions) because of their self-lubricating character e.g., dry sliding bearings [2]. Polymer-based systems are applied in medicine e.g., prostheses of human joints [3]. Applications of polymers in technology stimulate development of specific constitutive relations for friction including the material’s microstructure and its evolution. Descriptions of polymer friction should consider friction anisotropy and heterogeneity i.e., a dependence on sliding directions and positions on sliding trajectories.

**Figure 1:**
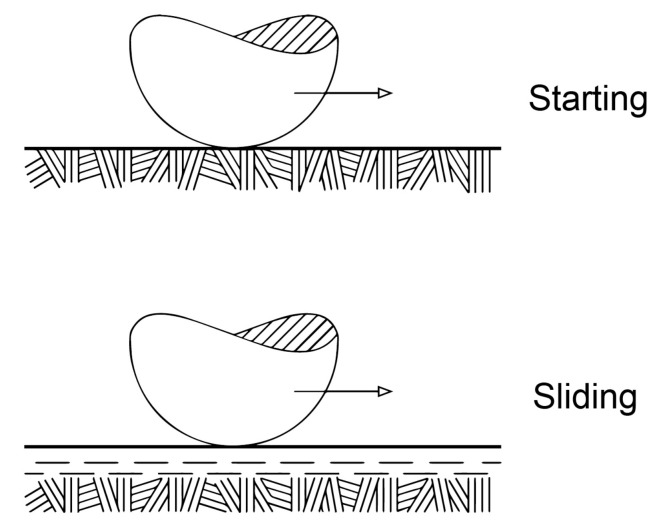
Schematic illustration of reorientation process of macromolecules on boundaries of certain solid polymers induced by sliding and friction.

There are various reasons of friction anisotropy and heterogeneity on boundaries of polymer solids, e.g., in polymer composites, semicrystalline polymers, and self-assembled and self-lubricating polymers. In composites consisting of components of different materials, external boundaries have mosaic structures, since different components are exposed at different points on the boundaries [4,5,6]. Polymers undergo morphological transformations, and they can transform from an amorphous phase into a semicrystalline phase [7,8]. The regular intrinsic structures (ordered individual chain segments) are in the semicrystalline phase. This causes anisotropic friction on external boundaries of polymer semicrystals [9]. In self-assembled and self-lubricating polymers, the sliding motion and friction induce microstructural changes in the surface and near-surface material [2,3]. Initially the macromolecules are randomly oriented, but under sliding and friction the molecular chains can be aligned in one direction [10], so that the microstructure may be highly anisotropic (Figure 1). This is responsible for significant changes of friction [3,11]. The sliding induced self-organization effects in the near-surface region were observed in other materials e.g., dislocations in copper [12]. On boundaries of solids, dry friction is also dependent on a surface roughness. An oriented surface roughness can be produced in the process of material machining [13,14]. Asymmetric surface roughness causes asymmetric friction e.g., smooth forward sliding and rough backward sliding.

**Figure 2:**
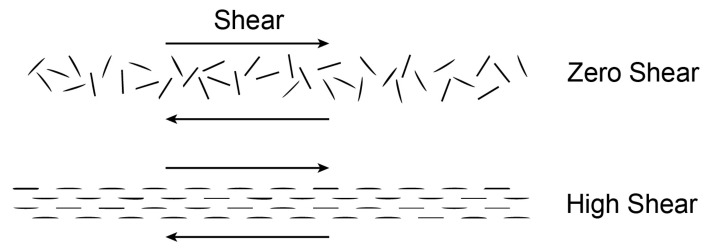
Schematic illustration of transition from random (isotropic) microstructure to oriented (anisotropic) microstructure inside polymer materials caused by large external loads (shear stresses).

Friction phenomena inside polymer materials are equally important as well as on external boundaries of polymer solids. Macromolecular polymers are repeated combinations of numerous simple chemical molecules (monomers) produced by a cyclic repetition in the fabrication process. Usually randomly distributed macromolecules do not have a specific orientation, but polymers are macromolecular materials with evolving properties. The evolution of polymer microstructure can be induced by action of large external loads (tension, shear) or high temperatures, and it has the following two forms: (a) unraveling of the molecular chains constituting the polymeric network, (b) relative motion of the unraveled chains or their segments through the surrounding moving macromolecules. In other words, large deformations initiate the orientation process of macromolecules [15,16,17]. Total macromolecules can be oriented along directions of the applied loads (or along streamlines of the fluid), so that the microstructure may be anisotropic (Figure 2). The macromolecules inside polymer materials move one against other, and a resistance to motion of macromolecules can be described with the aid of friction laws [18,19]. The motion resistance (friction) with anisotropy effects inside materials occurs in polymer melts, gels, solutions and liquid-crystals. The molecular chains move with highly anisotropic friction, i.e., with low friction for motions parallel to the chain and high friction for transverse motions.

The purpose of this study is to include microstructure evolution effects in friction equations for polymers on external boundaries of solids and inside materials. This is realized with the aid of anisotropic and nonhomogeneous friction models including useful additional independent variables. The paper is organized as follows: in a first step, friction on boundaries of solids is investigated (Section 2); in a second step, friction inside the polymer materials is analyzed (Section 3). Section 2.1 and Section 2.2 report shortly author’s anisotropic and nonhomogeneous dry friction models published in [20,21,22,23,24,25]. In Section 2.3 and Section 2.4, an example of anisotropic and heteogeneous dry friction is analyzed in the case of Archimedean spiral trajectory of a polymer pin sliding on a disc. Section 3.1 and Section 3.2 describe shortly known micromechanical models of individual polymer macromolecules, their kinematics inside materials and Langevin motion equation. Friction anisotropy inside the polymer materials is considered with the aid of various friction laws, viscous (Section 3.3) and dry (Section 3.4 and Section 3.5). In Section 3.4 and Section 3.5 two application examples of anisotropic friction are considered: a disc-like macromolecule model under conditions of spinning and sliding, a long macromolecule model under conditions of snake-like sliding. The application examples show how author’s friction models can be used to derive equations for friction forces in the given cases.

## 2. Models of Polymer Friction on Boundaries of Solids

### 2.1. Dry Friction Models with Additional Variables

Models of friction on external boundaries of polymer solids describe friction of polymers rubbing against another or against boundaries of different materials. In the literature, the model of dry friction or its contemporary modifications are used [5,13]. The microstructure evolution on boundaries of solid polymers is not included in these models.

**Figure 3:**
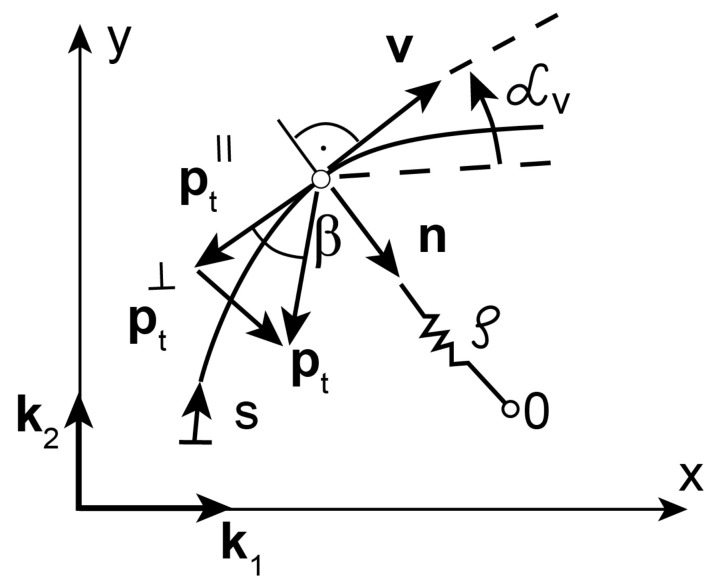
Sliding trajectory (s) of a material point in a plane with friction, friction force vector ($p_{t}$) with tangent and normal components ($p_{t}^{\|},p_{t}^{⊥}$), the angle of inclination (β), and independent variables of the friction force models; the tangent and normal unit vectors (**v**, **n**), the oriented angle of the sliding velocity ($\alpha_{v}$), and the radius of curvature (ρ) of the trajectory.

Macromolecular materials require specific constitutive relations for friction. Proposed in this study constitutive models of dry friction on boundaries of polymer solids are based on the phenomenological approach with additional variables that describe microstructural effects. The similar research methodology is used in advanced models of plasticity with internal (hidden) state variables [26]. The following quantities are taken as independent variables of dry friction models: unit vectors and oriented angles of sliding velocities, radii of curvature, and unit normal vectors of sliding trajectories (Figure 3).

#### 2.1.1. Dependence of the Friction Force on the Sliding Direction

Dependence of the friction force on the sliding direction is described with the aid of the unit vector **v** of the sliding velocity ${\dot{u}}_{t}$ (Figure 3), i.e.,

(1) $$v=\frac{{\dot{u}}_{t}}{{|\dot{u}}_{t}|}\;,\;\;\;|v|=1\;.$$

Taking into account Coulomb friction law, the tangent component $p_{t}$ of the contact force (the friction force vector) is defined as the function of the normal contact pressure $p_{n}$ and the unit vector **v**

(2) $$p_{t}=p_{t}\left(p_{n},v\right)=-|p_{n}|f\left(v\right)\;.$$

The friction force vector is oppositely directed to the sliding velocity.

Anisotropic friction for any sliding direction **v** is described by: the anisotropic friction coefficient $\mu_{\alpha }$ and the angle of friction force inclination β, i.e.,

(3) $$\mu_{\alpha }=\frac{1}{|p_{n}|}|p_{t}|\;,\;\;\;\;\;sin\beta =\frac{p_{t}\cdot n}{|p_{t}|}\;,$$

where, β∈[−π/2,π/2], **n** is the unit vector normal to the sliding trajectory. Coefficients of the friction force components collinear with the sliding direction, and normal to the sliding direction (Figure 3) are given by

(4) $$\mu_{\alpha }^{\|}=-\frac{1}{|p_{n}|}p_{t}\cdot v\;,\;\;\;\;\;\mu_{\alpha }^{⊥}=\frac{1}{|p_{n}|}p_{t}\cdot n\;.$$

Tangent and normal components of the friction force vector $p_{t}$ are as follows

(5) $$p_{t}^{\|}=-\mu_{\alpha }^{\|}|p_{n}|v\;,\;\;\;p_{t}^{⊥}=\mu_{\alpha }^{⊥}|p_{n}|n\;.$$

With the aid of the unit vector **v** as the independent variable, we extend Equation (2) including friction anisotropy [20]. The following single-term polynomial with respect to the sliding velocity unit vector **v** is taken as the linear model of anisotropic fiction

(6) $$p_{t}=-|p_{n}|C_{1}v=p_{t}^{i}k_{i}\;,$$

(7) $$C_{1}=C^{ij}k_{i}⊗k_{j}\;,\;\;i,j=1,2$$

where, $C^{ij}$ are coefficients of the second-order friction tensor $C_{1}$, $\left\{k_{1},k_{2}\right\}$ is an orthogonal basis of unit vectors in the reference system (Figure 3). The friction anisotropy is defined with the aid of the second-order tensor $C_{1}$ with constant coefficients. The matrix representation of the tensor $C_{1}$ has four coefficients

(8) $$\left[C_{1}\right]=\left[\begin{array}{cc} C^{11} & C^{12}\\ C^{21} & C^{22} \end{array}\right]\;.$$

The linear Equation (6) describes the friction cones with circular and elliptical shapes of cross-sections [20].

The following polynomial function of the sliding velocity unit vector **v** is the nonlinear model of anisotropic friction

(9) $$p_{t}=-|p_{n}|\left[C_{1}v+C_{2}\left(v⊗v⊗v\right)+\ldots \right]\;,$$

(10) $$C_{2}=C^{ijkl}k_{i}⊗k_{j}⊗k_{k}⊗k_{l}\;,\;\;i,j,k,l=1,2$$

where, $C_{2}$ is the fourth-order friction tensor. The representation of the fourth-order tensor $C_{2}$ is given by 16 coefficients as follows

(11) $$\left[C_{2}\right]=\begin{array}{cc} & \begin{array}{cccc} \;11\; & \;22\; & \;21\; & \;12\; \end{array}\\ \begin{array}{c} 11\\ 22\\ 21\\ 12 \end{array} & \left(\begin{array}{cccc} C^{1111} & C^{1122} & C^{1121} & C^{1112}\\ C^{2211} & C^{2222} & C^{2221} & C^{2212}\\ C^{2111} & C^{2122} & C^{2121} & C^{2112}\\ C^{1211} & C^{1222} & C^{1221} & C^{1212} \end{array}\right) \end{array}.$$

In the second term in Equation (9) as independent variable is taken the third-order tensor composed by the tensor products of the unit vectors **v**. Other higher order friction tensors can be included in Equation (9). The nonlinear Equation (9) describes the friction cones with complex shapes of the cross-sections (not only circular and elliptical shapes) [20].

#### 2.1.2. Dependence of the Friction Force on the Oriented Angle

Asymmetric surface roughness causes asymmetry of friction, i.e., variations in friction when one slides forward and then one slides backward (smooth and rough sliding). This is the so-called directionally asymmetric friction. The asymmetric friction description must include the sense of the sliding direction. Due to this we introduce the oriented angle (Figure 3) as the independent variable in friction equations [21,22]. Therefore, the friction tensors in the linear and nonlinear friction equations can depend on the oriented angle

(12) $$C_{i}=C_{i}\left(\alpha_{v}\right)\;,\;\;\;\alpha_{v}\in \left[0,2\pi \right]\;,\;\;i=1,2,3\ldots ,n$$

where, $\alpha_{v}$ is the measure of the oriented angle between the reference direction in the contact surface (e.g., *x*-axis) and the sliding direction **v** (Figure 3). The friction tensors in Equation (12) have nonconstant coefficients.

The friction tensors are assumed to be trigonometrical polynomials of the variable $\alpha_{v}$ as follows

(13) $$C_{i}\left(\alpha_{v}\right)=C_{i0}+C_{i1}cos\left(n_{i}\alpha_{v}\right)+C_{i2}sin\left(m_{i}\alpha_{v}\right)\;,\;\;\;n_{i},m_{i}=0,1,2,3,\ldots$$

where, $C_{il}$ (i=1,2,…,n;l=0,1,2) are tensors with constant coefficients. If we restrict Equation (13) to the second-order friction tensors, then the friction force vector has the following form

(14) $$p_{t}=-|p_{n}|\left[C_{10}+C_{11}cos\left(n_{1}\alpha_{v}\right)+C_{12}sin\left(m_{1}\alpha_{v}\right)\right]v\;.$$

If the parameters $n_{1},\;m_{1}$ are odd numbers, then Equation (14) describes non-centrosymmetric anisotropic friction. The cross-section of the friction cone has no center of symmetry in this type of friction [21,22]. The tensors $C_{10},C_{11},C_{12}$ with constant coefficients can be assumed to be arbitrary.

#### 2.1.3. Dependence of the Friction Force on the Sliding Trajectory Curvature

Dependence of the friction force on the position in the sliding trajectory is described with the aid of the radius of curvature ρ and the unit normal vector **n** (Figure 3). They are the local parameters of the trajectory useful to define friction heterogeneity [23,24,25]. According to the Frenet–Serret first formula, the first derivative of the unit vector **v** with respect to the parametrization *s* of the trajectory is given by

(15) $$\frac{dv}{ds}=\frac{n}{\rho }\;,$$

where, *s* is the one-dimensional parameterization of the sliding trajectory, **n** is the unit vector normal to the trajectory, n·v=0,∣n∣=1. Then, the friction force vector is defined as the following function

(16) $$p_{t}=p_{t}\left(p_{n},v,\frac{n}{\rho }\right)=-|p_{n}|f\left(v,\frac{n}{\rho }\right)\;.$$

The constitutive equation of the heterogenous friction force has two independent variables, i.e., the sliding velocity unit vector **v** and its derivative d**v**/ds.

In the first-order formulation, the heterogeneous friction force vector is defined by the sum of two single-term polynomials

(17) $$p_{t}=-|p_{n}|\left(C_{1}v+E_{1}n\frac{1}{\rho }\right)\;,$$

(18) $$E_{1}=E^{ij}e_{i}⊗e_{j}\;,\;\;\left\{e_{1},e_{2}\right\}\equiv \left\{v,n\right\}\;,\;\;i,j,=1,2$$

Two second-order tensors $C_{1}$ and $E_{1}$ describe frictional anisotropy and heterogeneity effects induced by the sliding motion and friction. The tensor $E_{1}$ is defined with the aid of the unit vectors tangent and normal to the sliding trajectory {**v**, **n**} at the given point of the trajectory.

The contraction of the tensor $E_{1}$ and the vector of the independent variable n/ρ (Equation (17)) gives the following

(19) $$E_{1}\frac{n}{\rho }=\left(E^{11}v⊗v+E^{12}v⊗n+E^{21}n⊗v+E^{22}n⊗n\right)\frac{n}{\rho }=\frac{E^{12}}{\rho }v+\frac{E^{22}}{\rho }n\;.$$

In Equation (19), we have two components depending on the sliding trajectory curvature: (a) dissipative type component, i.e., the additional friction $\left(E^{12}/\rho \right)$, and (b) a gyroscopic type component, i.e., the motion constraint $\left(E^{22}/\rho \right)$ normal to the trajectory. Therefore, the tensor $E_{1}$ describes constraints imposed on the motion in the directions tangent and normal to the sliding trajectory. The constraints are functions of the first power of the radii of curvature ρ of the sliding trajectory. The gyroscopic component can change essentially the sliding trajectory of the material point moving in a base plane; see [27].

Other higher order friction tensors can be included in Equation (17), i.e., fourth-order tensor $C_{2}$ see Equation (10), and by analogy fourth-order tensor $E_{2}$. In the case of $E_{2}$, the third-order tensor composed by the tensor products of the vectors **v** and n/ρ is taken as the independent variable. Friction equation with fourth-order tensors contains the radii of curvature ρ of the sliding trajectory raised to higher powers.

In the second-order formulation, the heterogeneous friction constitutive equation is given by the following polynomial

(20) $$p_{t}=-|p_{n}|\left\{C_{1}v+C_{2}\left(v^{3}\right)+E_{1}n\frac{1}{\rho }+E_{2}\left[\left(v^{2},n\right)\frac{1}{\rho }+\ldots +\left(v,n^{2}\right)\frac{1}{\rho^{2}}+\ldots +\left(n^{3}\right)\frac{1}{\rho^{3}}\right]\right\}\;.$$

The notation of the components of the third-order tensor used in Equation (20) as the independent variable of the friction force equation is as follows

(21) $$\left(v^{p},n^{q}\right)\frac{1}{\rho^{q}}\equiv \left(\underset{p\;copies}{\underbrace{v⊗\ldots ⊗v}}⊗\underset{q\;copies}{\underbrace{n⊗\ldots ⊗n}}\right)\frac{1}{\rho^{q}}\;,$$

(22) p=0,1,2,3,q=0,1,2,3,(p+q)=3.

Index 0 means—“zero copies” of the vectors **v** or n/ρ in the tensor products Equation (21). The fourth-order tensor $C_{2}$ defines friction anisotropy and heterogeneity. The contraction of the tensor $E_{2}$ and the third-order tensor as the independent variable in Equation (20) gives the following

$$E_{2}\left[\left(v⊗v⊗n\right)\frac{1}{\rho }+\ldots +\left(v⊗n⊗n\right)\frac{1}{\rho^{2}}+\ldots +\left(n⊗n⊗n\right)\frac{1}{\rho^{3}}\right]=$$

$$=[\left(E^{1112}+E^{1211}+E^{1121}\right)\frac{1}{\rho }+\left(E^{1122}+E^{1221}+E^{1212}\right)\frac{1}{\rho^{2}}+E^{1222}\frac{1}{\rho^{3}}]v+$$

(23) $$+[\left(E^{2112}+E^{2211}+E^{2121}\right)\frac{1}{\rho }+\left(E^{2122}+E^{2221}+E^{2212}\right)\frac{1}{\rho^{2}}+E^{2222}\frac{1}{\rho^{3}}]n\;.$$

The tensor $E_{2}$ describes the additional friction (the tangent component) and the motion constraint (the normal component), they depend on first, second, and third powers of the radii of curvature ρ of the sliding trajectory. By including second and third powers of ρ, we have the possibility to control an increase degree of the additional friction with respect to an increase of the radius of curvature. In other words, we have the possibility to control the coefficient evolution in sliding along the trajectory.

### 2.2. Thermodynamic Restrictions of the Friction Models

The proposed friction equations satisfy the axiom of objectivity. The material objectivity means that the friction force Equation (16) of the scalar $p_{n}$, the unit vector **v** and the vector n/ρ must be form-invariant with respect to any orthogonal transformation from the full orthogonal group O, i.e.,

(24) $$p_{t}\left(p_{n},\mathrm{Rv},R\frac{n}{\rho }\right)=Rp_{t}\left(p_{n},v,\frac{n}{\rho }\right)\;,\;\;\;\forall R\in O\;.$$

where, **R** is the tensor of the orthogonal transformation, $R^{-1}=R^{T}$ and detR=±1.

From the Second Law of Thermodynamics, it follows that in every case of frictional contact a power of the friction force (i.e., the scalar product of the friction force vector and the sliding velocity vector) is nonpositive.

(25) $$p_{t}\cdot {\dot{u}}_{t}\leq 0\;,\;\;\;\forall {\dot{u}}_{t}\;.$$

The friction constitutive functions are assumed to satisfy the dissipation inequality (25) for any sliding motion. The condition of the dissipated energy restricts constants (friction tensors) in the friction constitutive equations.

### 2.3. Movements and Friction of the Polymer Pin in Pin-on-Disc Tests

Friction and wear of polytetrafluoroethylene (PTFE) and high density polyethylene (HDPE) are sensitive to the orientation of the molecular chains with respect to sliding directions [28,29,30,31]. In [28], the sliding trajectories of a polymer pin in the form of concentric circles were investigated in pin-on-disc tests. An increase of the radii of concentric circular trajectories, and the resulting increase of the reorientation of molecular chains in the pin sliding surface (Figure 1), were responsible for changes of friction and wear [28].

With the aid of a multidirectional tribometer of the type pin-on-disc [3], friction and wear were investigated in dependence on shapes and curvatures of sliding trajectories, which the ultra-high-molecular-weight polyethylene (UHMWPE) pin drew on the disc. By changing the shapes of the trajectories (line, circle, Archimedean spiral, double Fermat spiral, lemniscate of Bernoulli), friction and wear were dependent on a polymer pin position moving on the sliding trajectory for constant values of the normal pressure and the sliding velocity. Kinematics of sliding and friction initiate reorientation of macromolecules in the pin sliding surface (Figure 1), and they are responsible for the heterogeneity of friction and wear [3].

**Figure 4:**
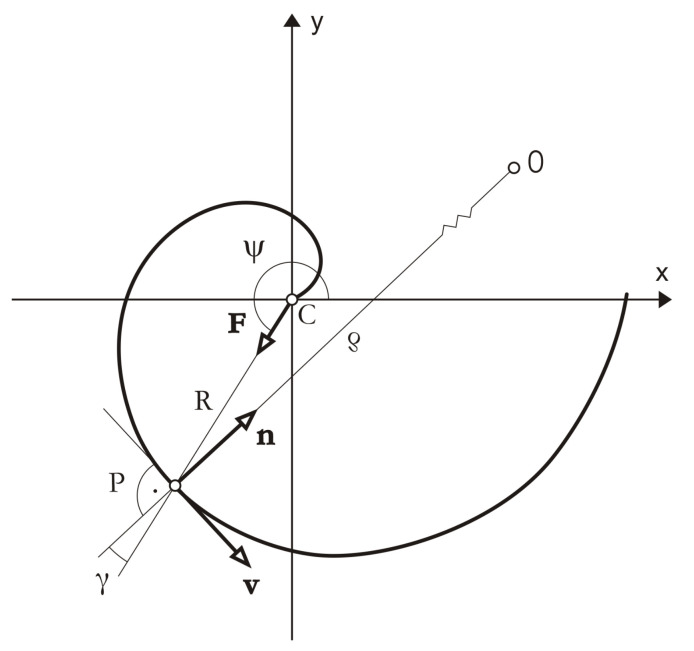
Archimedean spiral as sliding trajectory of the polymer pin.

As an illustrative example, we consider the sliding motion of a material point (the pin) in a sliding base plane (the disc) with the given sliding trajectory. The motion of the point in the reference system Cxy (Figure 4) is described by the following equation

(26) $$m\ddot{x}=F+p_{t}\;.$$

where, *m* is the mass of the material point, **x** is the position vector of the material point with respect to the reference system Cxy, $p_{t}$ is the friction force vector, **F** is the central force. The motion Equation (26) transformed to the local basis defined by the unit vectors tangent and normal to the sliding trajectory {**v**, **n**} (Figure 4) has the form as follows

(27) $$m\frac{dV}{dt}=F^{\|}+p_{t}^{\|}\;,$$

(28) $$m\frac{V^{2}}{\rho }=F^{⊥}+p_{t}^{⊥}\;.$$

where, *V* is the magnitude of the sliding velocity of the material point. The acceleration vector $\ddot{x}$ in Equation (26) is replaced by tangent and centrifugal components. In Equations (27) and (28) we need the tangent and normal components of the friction force vector with respect to the given sliding trajectory

(29) $$p_{t}=p_{t}^{\|}v+p_{t}^{⊥}n\;.$$

### 2.4. Archimedean Spiral as the Sliding Trajectory of the Polymer Pin

We consider the pin moving on Archimedean spiral specified as the sliding trajectory (Figure 4). Archimedean spiral in polar coordinates (R,ψ) is given by the radius *R* as the linear function of the angle ψ

(30) R(ψ)=aψ,ψ∈[0,∞],a=const

The radius of curvature ρ of the Archimedean spiral is a monotonically increasing function of the angle ψ as follows

(31) $$\rho \left(\psi \right)=\frac{a{\left(\psi^{2}+1\right)}^{3/2}}{\psi^{2}+2}\;.$$

The radii of curvature change smoothly in a large range of values ρ∈[0,∞]. With the aid of Equation (31) we have the possibility to determine positions on the spiral.

Let us consider heterogeneous friction in points on the Archimedan spiral taken as the sliding trajectory. It means that the friction force has various values in various points on Archimedean spiral. This refers to the microstructure evolving in sliding on Archimedean spiral trajectory in the case of some polymers [3]. There are two privileged sliding directions, i.e., along Archimedean spiral and along radii of curvature perpendicular to the spiral at the given point. The sliding along the spiral can occur with the lowest resistance to motion, and it can have the greatest resistance in the direction perpendicular to the spiral.

We assume that unit vectors $k_{1}$ and $k_{2}$ used in the definitions of the friction tensors are tangent and normal to Archimedean spiral respectively. Since the unit vectors {**v**, **n**} describe the moving reference frame whose origin is located at the point *P* (Figure 4), they replace unit vectors $k_{1}$ and $k_{2}$ in the definitions of friction tensors. Depending on the position on Archimedean spiral, the unit vectors change their orientations with respect to the reference system Cxy.

Taking into account the nonlinear friction equations, see Equations (9) and (20), we propose the following isotropic second-order tensor $C_{1}$ with one coefficient and orthotropic fourth-order tensor $C_{2}$ with two coefficients

(32) $$C_{1}=C^{11}\left(k_{1}⊗k_{1}+k_{2}⊗k_{2}\right)=C^{11}1\;,$$

(33) $$C_{2}=C^{1111}k_{1}⊗k_{i}⊗k_{1}⊗k_{i}+C^{2222}k_{2}⊗k_{i}⊗k_{2}⊗k_{i}\;,\;\;\;i=1,2$$

(34) $$\left\{k_{1},k_{2}\right\}\equiv \left\{v,n\right\}\;.$$

where **1** is the second-order unit tensor. The representation matrices of the isotropic friction tensor $C_{1}$ and orthotropic friction tensor $C_{2}$ are as follows

(35) $$\left[C_{1}\right]=\left[\begin{array}{cc} C^{11} & 0\\ 0 & C^{11} \end{array}\right]\;,$$

(36) $$\left[C_{2}\right]=\left[\begin{array}{cccc} C^{1111} & 0 & 0 & 0\\ 0 & C^{2222} & 0 & 0\\ 0 & 0 & C^{2222} & 0\\ 0 & 0 & 0 & C^{1111} \end{array}\right]\;.$$

Let us consider the second-order formulation Equation (20) of the heterogeneous friction force. We assume that the tensor $E_{1}$ has one coefficient and the tensor $E_{2}$ has two coefficients. The tensors $E_{1}$ and $E_{2}$ and their matrix representations are defined by

(37) $$E_{1}=E^{22}n⊗n\;,$$

(38) $$E_{2}=E^{2222}n⊗n⊗n⊗n+E^{1212}v⊗n⊗v⊗n\;,$$

(39) $$\left[E_{1}\right]=\left[\begin{array}{cc} 0 & 0\\ 0 & E^{22} \end{array}\right]\;,$$

(40) $$\left[E_{2}\right]=\left[\begin{array}{cccc} 0 & 0 & 0 & 0\\ 0 & E^{2222} & 0 & 0\\ 0 & 0 & 0 & 0\\ 0 & 0 & 0 & E^{1212} \end{array}\right]\;.$$

In the second-order formulation Equation (20), the heterogeneous friction force vector $p_{t}$ for the sliding along Archimedean spiral has the tangent (dissipative) $p_{t}^{\|}v$ and normal (gyroscopic) $p_{t}^{⊥}n$ components given by

(41) $$p_{t}\left(\rho \right)=-|p_{n}|\left[\left(C^{11}+C^{1111}+\frac{E^{1212}}{\rho^{2}}\right)v+\left(\frac{E^{22}}{\rho }+\frac{E^{2222}}{\rho^{3}}\right)n\right]\equiv p_{t}^{\|}v+p_{t}^{⊥}n.$$

The coefficient of the dissipative friction force component $\mu_{\alpha }^{\|}$ changes its value in points on Archimedean spiral in accordance to the following relation

(42) $$\mu_{\alpha }^{\|}\left(\rho \right)=C^{11}+C^{1111}+\frac{E^{1212}}{\rho^{2}}\;.$$

The last term in Equation (42) defines the additional friction (positive or negative), which depends on the second power of the spiral curvature ($1/\rho^{2}$). By including the second power of ρ we can control the coefficient evolution in sliding along the spiral, e.g., slow increase of the additional friction with respect to the increase of the radius of curvature.

The coefficient of the gyroscopic friction force component is given by

(43) $$\mu_{\alpha }^{⊥}\left(\rho \right)=\frac{E^{22}}{\rho }+\frac{E^{2222}}{\rho^{3}}\;.$$

It depends on the radii of curvature ρ raised to powers 1 and 3.

The coefficients $C^{11},\;C^{1111},\;E^{1212}$ and the radius of curvature ρ are restricted by the Second Law of Thermodynamics [23,24,25].

The material parameters used in the friction equations can be determined directly from experiments, since proposed dry friction models on boundaries of polymer solids are based on the phenomenological approach.

### 2.5. Models of Elastomer Friction on Boundaries of Solids

Usually two independent components describe the energy dissipation during sliding on the external boundaries of elastomer solids. So called two-component elastomer friction models include adhesion and hysteresis effects [32,33,34]. From the mechanical point of view, adhesion is the phenomenon that occurs when a normal tensile force must be done to separate two surfaces from contact after being compressed together. Rabinowicz [35] proposed such phenomenological model that normal and tangent components of the adhesive force depend on the initially applied compressive force.

Very high hysteresis of bulk deformations takes place in elastomers [32,33,34]. The hysteresis loop (force-displacement curve) is observed during cyclic loading and unloading. From the molecular scale point of view, this is the internal energy dissipation process due to friction between elastomer macromolecules inside the material. The hysteresis effects are time dependent. A contribution to the friction force on the external boundary of the elastomer solid due to the hysteresis of deformations can be estimated by the friction relaxation. In accordance with this assumption, the hysteresis dependent friction component can be described by a dependence between the friction force and time, e.g., this friction force component can be exponentially time-dependent.

## 3. Models of Polymer Friction Inside Materials

### 3.1. Micromechanical Models of Polymer Macromolecules

**Figure 5:**
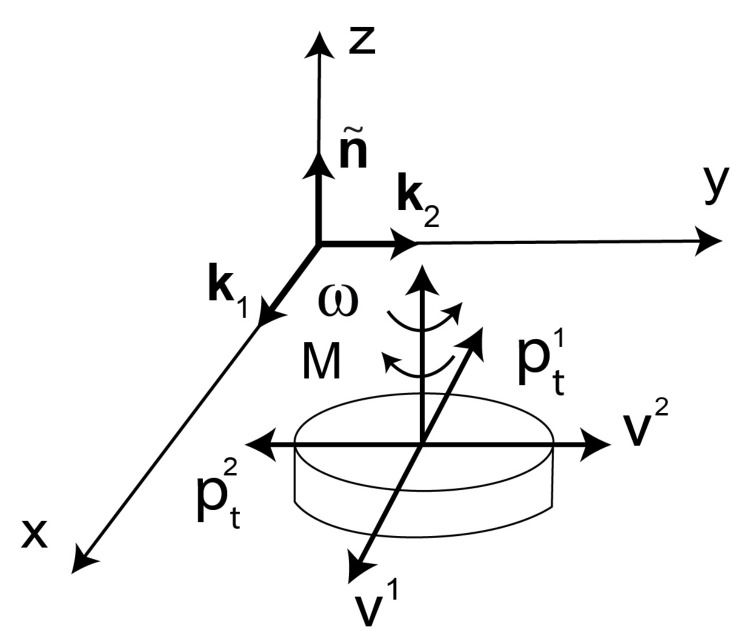
Combination of spinning and sliding of rigid disc on base plane in presence of anisotropic dry friction.

Polymers are modeled as assemblies of great number of isolated individual micro-elements composing the materials. In the frame of micromechanics various models of the individual molecular chains are proposed in the literature, e.g., bead-spring, bead-rod, elastic dumbbell model, reptation type tube, rod-like macromolecule, disc-like macromolecule (Figure 5). In the models, the individual macromolecules have idealized simple shapes. Furthermore, the individual molecular segments have specific modes of motion, e.g., sliding, rolling, and spinning.

Rouse model [36]—the macromolecule is represented by a chain of spherical beads connected by springs (or rods). The springs (rods) simulate elastic properties of the macromolecule. During the motion, the beads are affected by the surrounding molecules and the resistance to motion (friction) arises. The friction forces are located at bead centers since the beads have no volume.

De Gennes tube model [37]—the individual long macromolecule moves like a snake within a long and narrow tube. The virtual tube is formed by the surrounding molecular chains. The friction with anisotropy and asymmetry accompanies the snake-like motion.

Polymers in liquid crystal phase can form long rigid macromolecules similar to rods and flat rigid macromolecules similar to discs. During motions of rod-like and disc-like macromolecules the energy dissipation takes part since the resistance to motion between these objects is present. Two particular cases of the resistance to motion (friction) can be considered: (a) friction of rod-like macromolecules under rolling with and without slipping, (b) friction of disc-like macromolecules under spinning and sliding. In both cases, friction forces and friction couples are present.

From the mechanical point of view, polymers may have simultaneously viscous properties typical in fluids and elastic properties as in solids. In the literature, constitutive laws between stresses and strains in polymers are described with the aid of the following two approaches. (a) Phenomenological modeling. The models are extensions of the constitutive theories developed for metals and their alloys e.g., visco-elasiticity, visco-plasticity [26]. Melted polymers and polymer solutions are non-Newtonian complex fluids e.g., visco-elastic fluids. (b) Modeling based on micro-observations. A transition from micro level to the global behavior is formulated. Approaches based on micro-observations of polymers are called kinetic theories.

In this study, we use the similar research methodology and the motion resistance between molecular chains we describe: (a) in terms of an extension of constitutive ideas from bulk polymer materials, i.e., continuum-based models of friction, and (b) taking into account the micro-models and kinematics of the polymer chains, i.e., micromechanical models of friction. The resistance to motion of the macromolecules is described with the aid of friction laws [18,19].

The motion resistance of the moving molecular chains we consider with respect to the individual molecular model and kinematical properties of the chains. We assume that micromechanical models of polymer macromolecules are in contact with a hypothetical base plane in the presence of anisotropic dry friction [18]. Kinematics and anisotropic friction are taken into account in two illustrative examples: (a) spinning and sliding of the disc-like macromolecule model, and (b) snake-like sliding of the long macromolecule model.

### 3.2. Macromolecule Dynamics including Friction Anisotropy

In the given conditions, the macromolecules in polymers are independent kinematical elements. Usually large external loads (or high temperatures) can initiate the movements of the molecular chains one with respect to other. The motion of the macromolecules consists of translations and rotations. Due to this the macromolecule models have translational and rotational degrees of freedom. Furthermore, the macromolecules can change shapes under large external loads. Individual polymer macromolecules meet the resistance to motion with very high degree of friction anisotropy [38].

The macromolecule motions are liquid-like and Brownian type. In stochastic dynamics, Langevin equation of motion describes Brownian movements using Newton motion equation with an additional term including external random excitations (stochastically fluctuating forces) [16,18,19,38,39,40]. The viscous friction law and the anisotropic friction tensor describe the resistance to motion of the polymer chains. Motion of the *i*-th bead (i.e., the segment of the polymer macromolecule) follows the Langevin equation:

(44) $$m\frac{d^{2}r_{i}}{dt^{2}}=\left[B^{11}k_{1}⊗k_{1}+B^{22}\left(1-k_{1}⊗k_{1}\right)\right]\frac{dr_{i}}{dt}-f_{i}-F_{i}\;,$$

(45) $$1=k_{1}⊗k_{1}+k_{2}⊗k_{2}+\tilde{n}⊗\tilde{n}\;,\;\;\;B^{22}\gg B^{11}\;,$$

where, *m* is the mass of the bead, $r_{i}\left(t\right)$ is the bead coordinate (i.e., the position vector in 3D space), $f_{i}$ is the stochastically fluctuating force (Gaussian white noise), $F_{i}$ is other external force, $k_{1}$ and $k_{2}$ are the unit vectors tangent and normal to the particular chain segment in a horizontal plane, $\tilde{n}$ is the unit vector in the transverse direction, **1** is the unit tensor, $B^{11},B^{22}$ are friction coefficients for motions tangent and normal to the particular chain segment respectively (the lowest friction coefficient and the largest friction coefficient) [38,40]. The Langevin motion equation is the ordinary differential equation. Brownian dynamics simulations are used for study polymer macromolecule motions and for prediction of polymer behaviors.

In computational mechanics various numerical techniques are used to predict the behavior of polymers as molecular systems when subject to applied loads: Molecular Dynamics (MD), Discrete Element Method (DEM), Finite Element Method (FEM). A precise friction constitutive model is a key factor in numerical calculations.

Nanometer is the length scale of the polymer chain. Nanosecond is the time scale in dynamics of polymer chains. Due to this, trial-and-error method can be a way of identification of the material parameters used in the micromechanical models.

### 3.3. Continuum-Based Models of Friction Inside Materials

In the literature, the models of anisotropic viscous friction are used as laws of the motion resistance of the polymer macromolecules due to interactions with the neighboring macromolecules [38,40]. In the models, the friction force acting on the molecular chain depends on the velocity (the relative velocity at the point, since the macromolecules move relative to one another), and friction anisotropy is represented by the viscous friction tensor [15,16,17,19,39].

The anisotropic viscous friction in Equation (44) is described by the force of translational anisotropic viscous friction as follows

(46) $$p=-B\dot{u}\;,\;\;\;\dot{u}=\frac{dr_{i}}{dt}\;,$$

where, $\dot{u}$ is the translational relative velocity, B is the tensor of translational anisotropic viscous friction

(47) $$B=B^{ij}k_{i}⊗k_{j}\;,\;\;k_{3}\equiv \tilde{n}\;,\;\;i,j=1,2,3$$

The vectors of the friction force **p** and the velocity $\dot{u}$ have horizontal and transversal components with respect to the flow direction

(48) $$p=p_{t}+p_{n}\;,\;\;\;\dot{u}={\dot{u}}_{t}+{\dot{u}}_{n}\;.$$

The representation matrix of the anisotropic viscous friction tensor B in Equation (44) has two coefficients as follows

(49) $$\left[B\right]=\left[\begin{array}{ccc} B^{11} & 0 & 0\\ 0 & B^{22} & 0\\ 0 & 0 & B^{22} \end{array}\right]\;.$$

This friction is orthotropic in the horizontal plane and isotropic in the transverse direction.

In [40], rod-like microstrucures are modeled as Cosserat continuum, i.e., they have rotational degrees of freedom [41]. In the case of Cosserat-like macromolecules, the resistance to rotational motion is described with the aid of a moment of rotational anisotropic viscous friction. An angular velocity vector is the independent variable in the rotational friction equation [40].

### 3.4. The Macromolecule Disc-like Model under Combination of Spinning and Sliding

Spinning is the angular motion about the normal axis to the disc and to the base plane. Spinning of the disc is described by the unit vector $\bar{\omega }$ of the angular velocity ω

(50) $$\bar{\omega }=\frac{\omega }{|\omega |}=\bar{\omega }\tilde{n}=\pm 1\tilde{n}\;.$$

The axis of rotation is called the spin center or the spin pole or the center of gravity (Figure 5). Let the position of the contact point P in the unit contact area dF with respect to the center of spin is defined by the vector $r=r^{i}k_{i}$ (*i* = 1,2). The scalar function $p_{n}\left(r\right)$ of the vector variable **r** describes arbitrary distribution of the normal pressure at the contact area *F* with arbitrary shape and dimensions. During spinning, the disc particles contacting with the base plane have translational velocities. The unit vector $\bar{v}$ of the translational velocity at an arbitrary point of contact is associated with the angular velocity unit vector $\bar{\omega }$ and is defined by the relation

(51) $$\bar{v}=\frac{\bar{\omega }\times r}{|\bar{\omega }\times r|}={\bar{v}}^{i}k_{i}\;,\;\;\;i=1,2$$

(52) $${\bar{v}}^{1}=-\frac{\bar{\omega }r^{2}}{\sqrt{{\left(r^{1}\right)}^{2}+{\left(r^{2}\right)}^{2}}}\;,\;\;\;{\bar{v}}^{2}=\frac{\bar{\omega }r^{1}}{\sqrt{{\left(r^{1}\right)}^{2}+{\left(r^{2}\right)}^{2}}}\;.$$

Let the friction force at point P of the unit contact area dF be defined by the linear equation of anisotropic friction Equation (6), i.e.,

(53) $$p_{t}\left(P\right)=-|p_{n}\left(r\right)|C_{1}\bar{v}=p_{t}^{i}\left(P\right)k_{i}\;,\;\;\;i=1,2$$

with the second-order friction tensor $C_{1}$ having four coefficients Equation (8). The components of the friction force $p_{t}\left(P\right)$ at the spin contact have the following forms

(54) $$p_{t}^{i}\left(P\right)=-|p_{n}\left(r\right)|C^{ik}\delta_{kj}{\bar{v}}^{j}=-|p_{n}\left(r\right)|H_{P}^{i}\bar{\omega }\;,\;\;\;i,j,k=1,2$$

(55) $$H_{P}^{1}=\frac{1}{\sqrt{{\left(r^{1}\right)}^{2}+{\left(r^{2}\right)}^{2}}}\left\{-C^{11}r^{2}+C^{12}r^{1}\right\}\;,$$

(56) $$H_{P}^{2}=\frac{1}{\sqrt{{\left(r^{1}\right)}^{2}+{\left(r^{2}\right)}^{2}}}\left\{-C^{21}r^{2}+C^{22}r^{1}\right\}\;.$$

where, $\delta_{kj}$ is the Kronecker delta (operator). The moment of the friction force $p_{t}$ at point P with respect to the center of the disc (Figure 5) is defined by the vector

(57) $$M_{P}=r\times p_{t}=-|p_{n}\left(r\right)|H_{P}^{3}\bar{\omega }\tilde{n}\;,$$

(58) $$H_{P}^{3}=\frac{1}{\sqrt{{\left(r^{1}\right)}^{2}+{\left(r^{2}\right)}^{2}}}\left\{C^{11}{\left(r^{2}\right)}^{2}+C^{22}{\left(r^{1}\right)}^{2}-\left(C^{12}+C^{21}\right)r^{1}r^{2}\right\}\;.$$

Using the integration, the resultant friction force and the resultant friction moment are calculated. The resultant vectors of friction forces at the spin contact with the area *F*, and with arbitrary shape of the disc can be written as

(59) $$p_{t}=\int_{F}p_{t}\left(P\right)dF=p_{t}^{i}k_{i}\;,\;\;\;i=1,2$$

(60) $$M=\int_{F}M_{P}dF=M\tilde{n}\;,\;\;\;P\subset F$$

where the components of the vectors are as follows

(61) $$p_{t}^{i}=\int_{F}p_{t}^{i}\left(P\right)dF=-\bar{\omega }\int_{F}|p_{n}\left(r\right)|H_{P}^{i}dF=-\bar{\omega }H^{i}\;,\;\;\;i=1,2$$

(62) $$M=\int_{F}M_{P}dF=-\bar{\omega }\int_{F}|p_{n}\left(r\right)|H_{P}^{3}dF=-\bar{\omega }H^{3}\;.$$

The quantities defined by the formulas

(63) $$H^{l}=\int_{F}|p_{n}\left(r\right)|H_{P}^{l}dF\;,\;\;\;l=1,2,3$$

are called the friction characteristics.

Let us assume that the normal pressure is distributed uniformly at all contact points, i.e., $p_{n}\left(r\right)=p_{n}=const$. Such assumption is acceptable for the contact of rigid bodies. The case of contacting deformable solids is very different. In technological applications of contacting deformable bodies, the contact pressure is not uniformly distributed at the contact area. Often in technology we have Hertz contact pressure distribution, i.e., the pressure is semielipsoidally distributed over the circular or elliptical contact area. In the case of uniform contact pressure the friction characteristics have the following forms

(64) $$H^{1}=|p_{n}|\left(-C^{11}A_{1}+C^{12}A_{2}\right)\;,$$

(65) $$H^{2}=|p_{n}|\left(-C^{21}A_{1}+C^{22}A_{2}\right)\;,$$

(66) $$H^{3}=|p_{n}|\left[C^{11}A_{3}+C^{22}A_{4}-\left(C^{12}+C^{21}\right)A_{5}\right]\;,$$

(67) $$A_{1}=\int_{F}\frac{r^{2}}{r}dF\;,\;\;\;A_{2}=\int_{F}\frac{r^{1}}{r}dF\;,\;\;\;A_{3}=\int_{F}\frac{{\left(r^{2}\right)}^{2}}{r}dF\;,$$

(68) $$A_{4}=\int_{F}\frac{{\left(r^{1}\right)}^{2}}{r}dF\;,\;\;\;A_{5}=\int_{F}\frac{r^{1}r^{2}}{r}dF\;,\;\;\;r=\sqrt{{\left(r^{1}\right)}^{2}+{\left(r^{2}\right)}^{2}}\;.$$

If the contact area *F* has two orthogonal axes of symmetry and the point of intersection of the axes is the spin center, then $A_{1}$ and $A_{2}$ are equal to zero. Thus, the total friction force $p_{t}$ in contact with the base plane during spinning about the spin center is equal to zero.

Let us consider two friction cases of the base plane: (a) with isotropic friction, (b) with orthotropic friction, i.e.,

(69) $$\left[C_{1}\right]=\left[\begin{array}{cc} C^{11} & 0\\ 0 & C^{11} \end{array}\right]\;,$$

(70) $$\left[C_{1}\right]=\left[\begin{array}{cc} C^{11} & 0\\ 0 & C^{22} \end{array}\right]\;.$$

In these cases, the shape and dimensions of the contact area *F* are constant during spinning. Then, the friction characteristics are defined by the following formulas for the contact with isotropic friction Equation (69)

(71) $$H^{1}=-C^{11}|p_{n}|A_{1}\;,\;\;\;H^{2}=C^{11}|p_{n}|A_{2}\;,\;\;\;$$

(72) $$H^{3}=C^{11}|p_{n}|\left(A_{3}+A_{4}\right)\;.$$

For the contact with orthotropic friction Equation (70), the friction characteristics are determined by Equations (64)–(66), where $C^{12}=C^{21}=0$.

We assume that the contact area *F* of the disc has the circular shape with the radius *a*, and it rotates about the center of the disc; then, the quantities $A_{i}\;\left(i=1,2,\ldots ,5\right)$ are as follows

(73) $$A_{3}=A_{4}=\frac{1}{3}\pi a^{3}\;,$$

(74) $$A_{1}=A_{2}=A_{5}=0\;.$$

The vectors of the friction forces and their moments for the contact of the disc and the base plane with isotropic friction Equation (69) are given by

(75) $$p_{t}=0\;,\;\;\;M=-\frac{2}{3}C^{11}|p_{n}|\pi a^{3}\bar{\omega }\;,$$

and for the orthotropic friction Equation (70) of the base plane we have

(76) $$p_{t}=0\;,\;\;\;M=-\frac{1}{3}\left(C^{11}+C^{22}\right)|p_{n}|\pi a^{3}\bar{\omega }\;.$$

We consider coupled kinematics when the disc-like macromolecule participate simultaneously in sliding and spinning motions (Figure 5). Translation with the velocity unit vector **v** and spinning about the axis normal to the contact with velocity unit vector $\bar{\omega }$ describe the coupled motions of the disc. From the dynamical point of view, the disc is considered as the system with three degrees of freedom, i.e., two degrees of freedom for sliding movements and one degree of freedom for spinning. The friction force field at the contact area *F* is described by the resultant vector $p_{t}$ of the friction forces and the resultant vector **M** of the friction force moments with respect to the center of the disc. In accordance with the friction models, the linear mapping *L* exists between the resultant vectors of the friction forces and the unit vectors of the translational and spinning velocities, i.e.,

(77) $$\left[\begin{array}{c} p_{t}^{1}\\ p_{t}^{2}\\ M \end{array}\right]=\left[L\right]\left[\begin{array}{c} v^{1}\\ v^{2}\\ \bar{\omega } \end{array}\right]\;.$$

We consider the forms of the mapping *L* at the contact of the circular disc of the radius *a* and the constant normal pressure. In the case of isotropic friction Equation (69), we have

(78) $$\left[L\right]=-|p_{n}|\left[\begin{array}{ccc} \pi a^{2}C^{11} & 0 & 0\\ 0 & \pi a^{2}C^{11} & 0\\ 0 & 0 & \frac{2}{3}\pi a^{3}C^{11} \end{array}\right]\;,$$

and in the case of orthotropic friction Equation (70), we get

(79) $$\left[L\right]=-|p_{n}|\left[\begin{array}{ccc} \pi a^{2}C^{11} & 0 & 0\\ 0 & \pi a^{2}C^{22} & 0\\ 0 & 0 & \frac{1}{3}\pi a^{3}\left(C^{11}+C^{22}\right) \end{array}\right]\;.$$

Dynamics of rigid discs having elliptical shapes in the presence of anisotropic and asymmetric friction in the base sliding plane and under conditions of sliding and spinning were studied in [42].

### 3.5. The Long Macromolecule Model under Snake-like Sliding

In De Gennes tube model [37], friction anisotropy and friction asymmetry manifest as the snake-like motion of the long macromolecule. Due to this, one should include directional differences in sliding friction typical for snakes (and snake-like robots) [43]. The snake skin covered with snake scales has anisotropic friction properties. Furthermore, asymmetric surface micro-structure of the snake skin causes asymmetry of friction, i.e., higher friction for backward sliding, lower friction for forward sliding. The sliding in the transverse direction has higher friction compared to friction in the forward sliding. The friction descriptions of the snake skin must include the sliding direction and the sense of the sliding direction.

In this case, we use the noncentrosymmetric friction model Equation (14). The sliding direction **v** and the oriented angle $\alpha_{v}\in \left[0,2\pi \right]$ are the independent variables in friction equations [21,22]. Taking into account Equation (14), we assume the following friction force equation in the snake-like sliding of the long macromolecule

(80) $$p_{t}=-|p_{n}|\left[C_{10}+C_{11}cos\left(n_{1}\alpha_{v}\right)\right]v\;,$$

(81) $$\left[C_{10}\right]=\left[\begin{array}{cc} C_{0}^{11} & 0\\ 0 & C_{0}^{22} \end{array}\right]\;,\;\;\;\left[C_{11}\right]=\left[\begin{array}{cc} C_{1}^{11} & 0\\ 0 & C_{1}^{22} \end{array}\right]\;,\;\;\;n_{1}=1$$

(82) $$\left[v\right]={\left[v^{1}\;\;v^{2}\right]}^{T}={\left[cos\alpha_{v}\;\;sin\alpha_{v}\right]}^{T}$$

This snake-like friction model has four coefficients.

## 4. Conclusions

The polymer friction models are considered in terms of specific material’s microstructures, specific friction mechanisms, various friction laws, and different scales on external boundaries of solids and inside materials. Proposed models of polymer friction take into account: sliding directions (friction anisotropy), positions on sliding trajectories (friction heterogeneity), specific models of individual macromolecules, and their kinematics inside the polymer materials.

Main advantages of the proposed friction models are as follows: they are simple enough and they have a finite number of parameters (easy to determine in experiments), and they can be extended to other types of frictional anisotropy and nonhomogeneity important in polymers. Applied in this study, continuum mechanics principles are a powerful tool in the modeling of polymer friction.

The friction models on external boundaries of solid polymers can be used to simulate, predict, and improve the frictional behavior of polymeric component parts of mechanical systems, e.g., dry sliding bearings, sliding seals, transmission belts, car tires, prostheses of human joints, etc.

The friction models inside the polymer materials can be used in studies of macromolecule dynamics with friction effects, and they can have practical applications in simulation and optimization of properties of polymer melts, gels, solutions, and liquid crystals in fabrication processes of the chemical industry.

## Data Availability

Data is contained within the article.
